# Clinical and perioperative outcomes of abdominal wall reconstruction and panniculectomy in a single surgical procedure: experience from a high-complexity center in bogotá, Colombia

**DOI:** 10.1007/s10029-025-03415-7

**Published:** 2025-07-21

**Authors:** Carolina Riscanevo Bobadilla, Alejandro Lora Aguirre, Juan Pablo Ruiz, Neil Valentín Vega, Arnold José Barrios, Ana María Sastre, German Paez, David Duque, Camilo Mejia, Camilo Herrera, Luis Arturo Molina, María del Pilar Montilla

**Affiliations:** 1 Department of General Surgery, Fundación Universitaria Sanitas, Clínica Universitaria Colombia, Bogotá, D.C Colombia; 2 Abdominal Wall Group, Department of General Surgery, General Surgeon, Clínicas Colsanitas, Bogotá, D.C Colombia; 3 Department of Plastic Surgery, Clínica Universitaria Colombia, Bogotá, D.C Colombia; 4 Clínica Universitaria Colombia, Bogotá, D.C Colombia; 5 Fundación Universitaria Sanitas, Bogotá, D.C Colombia

**Keywords:** Abdominal wall, Ventral hernia, Abdominoplasty, Obesity, Surgical wound infection

## Abstract

**Purpose:**

Patients with obesity and a large pannus may benefit from the simultaneous performance of panniculectomy and ventral hernia repair. However, the literature reports conflicting results regarding an increased risk of surgical site events when both procedures are performed concurrently. This study aimed to describe the outcomes of patients undergoing simultaneous ventral hernia repair and panniculectomy.

**Methods:**

A retrospective single-arm cohort study was conducted, including consecutive patients who underwent abdominal wall reconstruction and panniculectomy during the same surgical procedure at a high-complexity center in Bogotá, Colombia, between 2014 and 2024. Demographic data, intraoperative variables, and postoperative outcomes were collected and analyzed.

**Results:**

A total of 171 patients underwent combined abdominal wall reconstruction and panniculectomy in a single surgical session. The mean age was 54 ± 12.8 years, and the average body mass index (BMI) was 27.77 ± 4.7. Male patients accounted for 26.3% of the cohort. The most common complications were seroma, hematoma, and skin dehiscence. The median hospital stay was 2 days (± 3.3). Early discharge with continued recovery through a home hospitalization program was achieved in 11.1% of the patients.

**Conclusion:**

Abdominal wall reconstruction combined with panniculectomy is a feasible surgical strategy that not only restores the dynamic functionality of the abdominal wall but also improves the patient’s aesthetic appearance. This study represents the first published experience in Latin America aiming to evaluate the safety, feasibility, and clinical benefits of this combined approach in real-world medical practice.

**Supplementary Information:**

The online version contains supplementary material available at 10.1007/s10029-025-03415-7.

## Introduction

In the United States, approximately 4 million laparotomies are performed annually [1]– [2]. Between 5% and 24% of these procedures result in incisional hernias [3]. Recurrence rates after surgical repair range from 30 to 54%, depending on factors such as hernia defect size, anatomical location, and associated patient comorbidities [4]. However, local data on the incidence of these hernias in Colombia is limited, making it difficult to accurately estimate the economic and social burden they represent in the country.

Abdominal wall hernias—particularly ventral hernias—are a common complication in patients who have undergone laparotomy. This risk is significantly increased in individuals who are overweight or obese due to sustained elevated intra-abdominal pressure and potential alterations in connective tissue integrity. Obesity, in particular, has been identified as an independent risk factor contributing to both the development and recurrence of primary hernias, with rates reported as high as 45% [5]. Excess body weight is also associated with reduced mobility, hygiene difficulties, and a higher predisposition to metabolic disorders and chronic infections, all of which negatively affect quality of life. Moreover, it correlates with increased surgical and medical complications, which tend to rise proportionally with the patient’s body fat percentage [6, 7].

There are several chronic abdominal wall conditions that present a challenge for abdominal wall reconstruction (AWR) surgery. These include lipodystrophy, sequelae of open abdomen management, loss of domain hernias, history of multiple hernia recurrences, parastomal hernias, abdominal wall tumors, and large hernia defects [8]. Such conditions require a comprehensive approach that addresses both functional and aesthetic aspects. Skin and subcutaneous tissue play a crucial role in the incidence of local surgical complications such as seroma, hematoma, surgical site infection, and mesh contamination or infection [8]. Achieving both functional and aesthetic outcomes improves treatment effectiveness and optimizes resource use [9].

Therefore, performing abdominal wall reconstruction and panniculectomy simultaneously in selected patients is becoming a viable option, as it minimizes the number of surgical interventions, provides better surgical exposure, facilitates the resection of poorly perfused tissue, reduces flap tension during repair, and improves both aesthetic outcomes and patient quality of life [8]. However, the literature has debated the role of concomitant panniculectomy during abdominal wall reconstruction due to concerns about increased operative time and surgical complications. It has been associated with a higher incidence of surgical site occurrences (SSOs), the need for reoperation, and longer hospital stays [5], raising questions about the advisability of performing both procedures at the same time. Variability in surgical teams, techniques used, and patient characteristics, among other factors, may contribute to these outcomes [10].

Managing such cases requires the involvement of multidisciplinary surgical teams, including specialists in plastic surgery, abdominal wall surgery, nutrition, physiatry, radiology, and others. It also demands additional efforts from healthcare institutions, insurers, and patients themselves to align the interests of all parties involved [8]. A comprehensive approach includes joint preoperative evaluation (medical board review, clarity of team roles), along with administrative and logistical support from institutions to implement standardized management protocols for these patients. There is evidence supporting the positive impact of these measures on surgical outcomes [11]. The purpose of this study was to describe the institutional experience of integrating abdominal wall reconstruction and panniculectomy in selected patients, in order to characterize outcomes associated with different surgical strategies and to identify key factors influencing the results.

## Methods and materials

### Study design and population

A retrospective, single-arm cohort study was conducted using a prospective database developed based on the experience of the abdominal wall reconstruction group. Consecutive selection of patients was performed from 2017 to 2024. A total of 171 patients who underwent abdominal wall reconstruction and panniculectomy during the same surgical procedure were identified at a high-complexity center in Bogotá, D.C., Colombia.

Patients who underwent procedures in the context of emergency surgery and those who were lost to follow-up were excluded from the study.

### Preoperative evaluation

Abdominal wall defects were classified according to the guidelines of the European Hernia Society [12]. Preoperative optimization of the patients was performed considering weight loss to achieve a BMI < 35; for this, patients were referred for evaluation by endocrinology, physical medicine, and clinical nutrition. In diabetic patients, priority was given to controlling serum glucose levels, setting a target for glycated hemoglobin < 7.5%, as well as smoking cessation at least one month before the procedure.

**Fig. 1:**
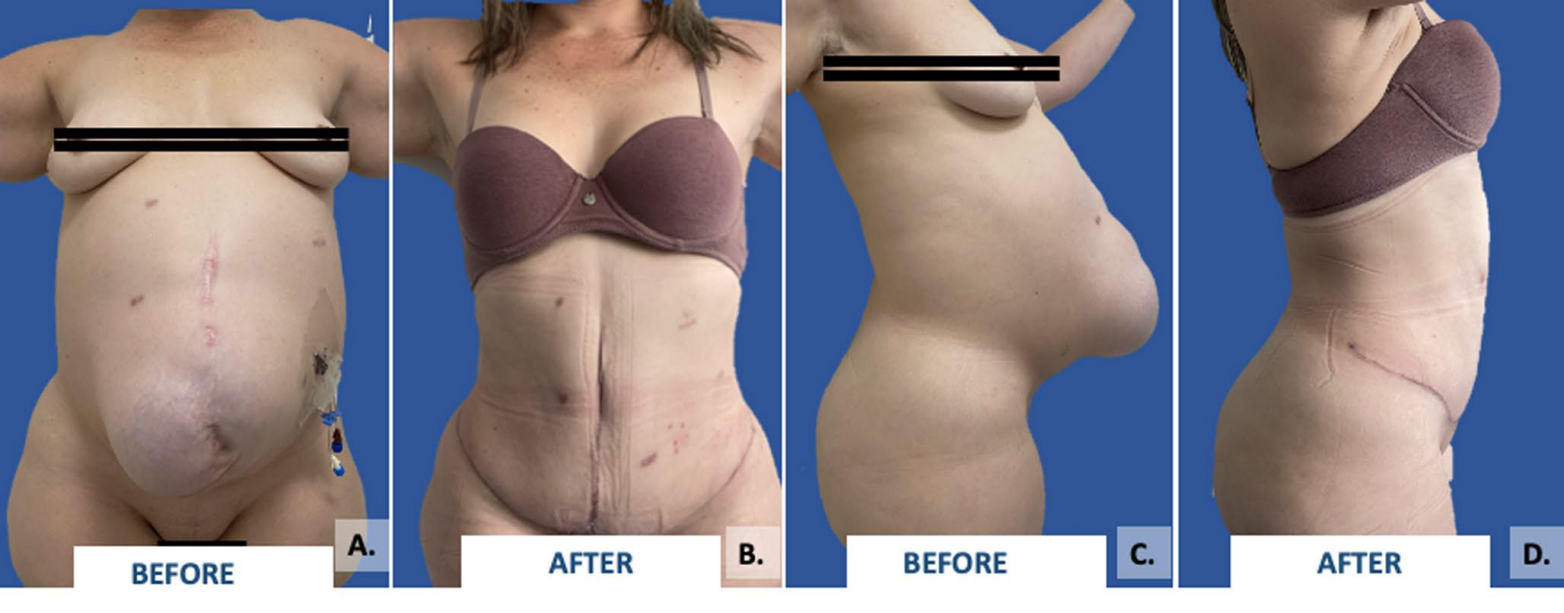
Patient with a complex ventral hernia (M1–M5, W3–14 cm). **(A)** Administration of progressive preoperative pneumoperitoneum to facilitate reintroduction of herniated viscera and expand abdominal domain. **(B)** Abdominal wall reconstruction performed using the posterior component separation technique (Rives-Stoppa) and horizontal panniculectomy to optimize both functional and aesthetic outcomes

Among the preoperative studies, all patients underwent dynamic computed tomography (CT) to characterize the hernia defect, the abdominal wall musculature, and to rule out other pathologies. Additionally, it allowed for volumetric analysis and planning of the surgical procedure. In patients with loss of domain, defined as an irreducible hernia where midline restoration cannot be performed primarily, and there is a risk of compartment syndrome when attempting closure [12]; In this study, an objective and standardized definition of “loss of domain” (LOD) was adopted, particularly relevant in the context of preoperative assessment using dynamic computed tomography. The Tanaka index was used, which defines LOD as a hernia sac volume greater than 20% of the total abdominal volume. In patients meeting this criterion, abdominal wall prehabilitation techniques were employed—such as progressive preoperative pneumoperitoneum and/or the administration of botulinum toxin (botox)—as illustrated in Fig. 1, with the aim of optimizing anatomical conditions prior to reconstructive surgery.

The procedures were carried out by the hernia and abdominal wall surgery group, with more than ten years of experience in collaborative work and standardized practices. The panniculectomy was performed by plastic surgeons. Panniculectomy consisted of the removal of excess skin and subcutaneous fatty tissue from the abdominal wall [8]. It was indicated when there was excess tissue greater than 4 cm in transverse diameter. This technique can be adapted to the individual needs of each patient through different approaches, such as vertical, horizontal, or fleur-de-lis panniculectomy. The selection of the approach was based on the clinical characteristics of the patient, the type of previous surgical intervention, and its impact on the restoration of the midline, performed by the treating plastic surgeon. A transverse panniculectomy was performed when the excess tissue was predominantly infraumbilical and distributed along the horizontal axis. The vertical technique was indicated for redundancy of the abdominal pannus in the horizontal plane. Meanwhile, the fleur-de-lis technique, which combines vertical and horizontal resections, was applied in cases with excess skin in both directions.

All patients were followed for 60 days postoperatively. Complication data were collected through standardized postoperative follow-up visits conducted at 2 weeks, 1 month, 3 months, and 6 months after surgery. Aesthetic and functional outcomes were assessed using clinical and observational criteria during postoperative follow-up visits. This evaluation included aspects such as recovery of abdominal posture and mobility, return to daily activities, presence of incisional hernias or abdominal wall weakness, postoperative pain, as well as abdominal wall symmetry and contour. Additionally, standardized pre- and postoperative photographs were used as a complementary tool to document the observed changes.

Complications were classified according to the Clavien-Dindo scale. Sociodemographic data and medical comorbidities were recorded, and intraoperative and postoperative variables were analyzed to assess outcomes in patients undergoing concomitant abdominal wall reconstruction and panniculectomy.

The hernia and abdominal wall surgery group, after a comprehensive analysis of the patient, their social context, and the complexity of the surgical procedure, determined their eligibility for the Home Care Program (HCP) implemented in the institution. This modality represents an extension of inpatient management, especially useful in addressing the imbalance between the supply and demand of hospital beds. The HCP is aimed at low-risk surgical patients without intraoperative complications and with the possibility of continuous follow-up, who are candidates for early discharge within the program. Conversely, patients over 70 years of age, with functional dependence, or who do not meet the inclusion criteria for the HCP are managed in the hospital setting during their postoperative period.

The procedures were performed by members of the hernia and abdominal wall surgery group in all cases—plastic surgeons and general surgeons—after patient discussion in a medical board meeting. The surgical techniques for abdominal wall reconstruction and panniculectomy (abdominoplasty) are standardized by the group. After hospital discharge, medical follow-up was carried out through telephone calls and in-person evaluations in the outpatient clinic.

During abdominal wall hernia repair using a retromuscular approach, the two most commonly employed techniques are the Rives and Rives-Stoppa methods.The Rives technique is based on a localized retromuscular dissection, which may be unilateral or bilateral, and is particularly indicated for medium-sized or infraumbilical hernias. In this approach, the mesh is placed directly over the posterior sheath of the rectus abdominis muscle without the need for extensive dissection. The aim is to reinforce the hernia defect through an anatomic repair with minimal disruption of the posterior plane. In contrast, the Rives-Stoppa technique is an extension of the original approach and involves a wide bilateral dissection of the retromuscular space, from the midline to the semilunar lines. This exposure allows access to both the Bogros space and the Retzius space, facilitating the placement of a large mesh with broad overlap of the hernia defect.

### Statistical analysis

The results of the analysis were presented using absolute and relative frequencies for qualitative variables, and central tendency and dispersion measures for quantitative variables, according to the normality test results. Associations between possible determinants of complications and hospital stay were evaluated using logistic regression analysis. A significance level of *p* < 0.05 was considered statistically significant.

## Results

A total of 171 patients were included, who underwent sequential abdominal wall reconstruction and panniculectomy in the same surgical time, with a mean age of 54 ± 12.8 years and an average BMI of 27.77 ± 4.7 kg/m2. Of these, 29.82% presented obesity. 73.7% (*N* = 126) of the patients were women. A history of smoking was present in 17.5% of patients, and they discontinued smoking for at least one month as part of the preoperative preparation.

Regarding the ASA classification, 73.9% of the patients were categorized as ASA II, and 27.7% as ASA III. Among the comorbidities, 26.3% had a history of hypertension, 15.2% had diabetes mellitus, and 3.5% had coronary artery disease. Additionally, 38% had a history of previous abdominal wall hernia repair. Indication for surgery to resect abdominal wall tumors was present in 10% (17/170) of the cases. Demographic results are included in Table. 1.

Concerning hernia characteristics, the mean defect area ranged between 10 ± 9.9 cm. Progressive preoperative pneumoperitoneum was indicated in 3.5% of the patients, and botulinum toxin use was applied in up to 29.5% of the patients.

**Table 1:** Demographic and clinical characteristics of the patients

Variables	*N* = 171
**Demographics**	
Male sex	45 (26.32%)
Age in years (mean ± SD)	54 (± 12.84)
**Medical History**	
Smoking	30 (17.54%)
COPD	10 (5.85%)
Dyslipidemia	27 (15.88%)
Diabetes mellitus	26 (15.29%)
Arterial hypertension	45 (26.32%)
Coronary artery disease	6 (3.51%)
History of radiotherapy	4 (2.35%)
Bariatric surgery	9 (5.29%)
Previous hernia	65 (38.01%)
Abdominal wall tumors	17 (10%)
History of abdominal surgery	142 (83.53%)
**Clinical Characteristics**	
**ASA Classification**	
I	16 (9.47%)
II	125 (73.96%)
III	28 (16.57%)
BMI (kg/m²)	27.77 (± 4.74)
Obesity	51 (29.82%)
**Iglesias Classification** [10]	
**Grade 1.** The pannus remains above the inguinal ligament.	11 (8.33%)
**Grade 2.** The overhanging pannus is below the inguinal ligament but does not surpass the upper third of the thigh.	102 (77.27%)
**Grade 3**. The overhanging pannus is located within the middle third of the thigh.	19 (14.39%)
**Grade 4.** The overhanging pannus is within the lower third of the thigh.	0
**Grade 5.** The overhanging pannus is below the knee.	0

**SD**: Standard Deviation; **COPD**: Chronic Obstructive Pulmonary Disease; **ASA**: American Society of Anesthesiologists; **BMI**: Body Mass Index

Table 2 summarizes the technical and surgical characteristics used in the patients. In all procedures, a synthetic polypropylene mesh was employed. An underlay position was used in 59.3% of cases, followed by an onlay position in 40%, and an inlay position in 0.6%. The inlay approach was used in one patient due to a coverage defect following resection of an endometrioma.

The Rives-Stoppa technique was performed in 23.3% of cases. The posterior sheath was closed using a 2/0 absorbable monofilament suture (polydioxanone). The mesh was shaped to fit the retrorectal space.

**Fig. 2:**
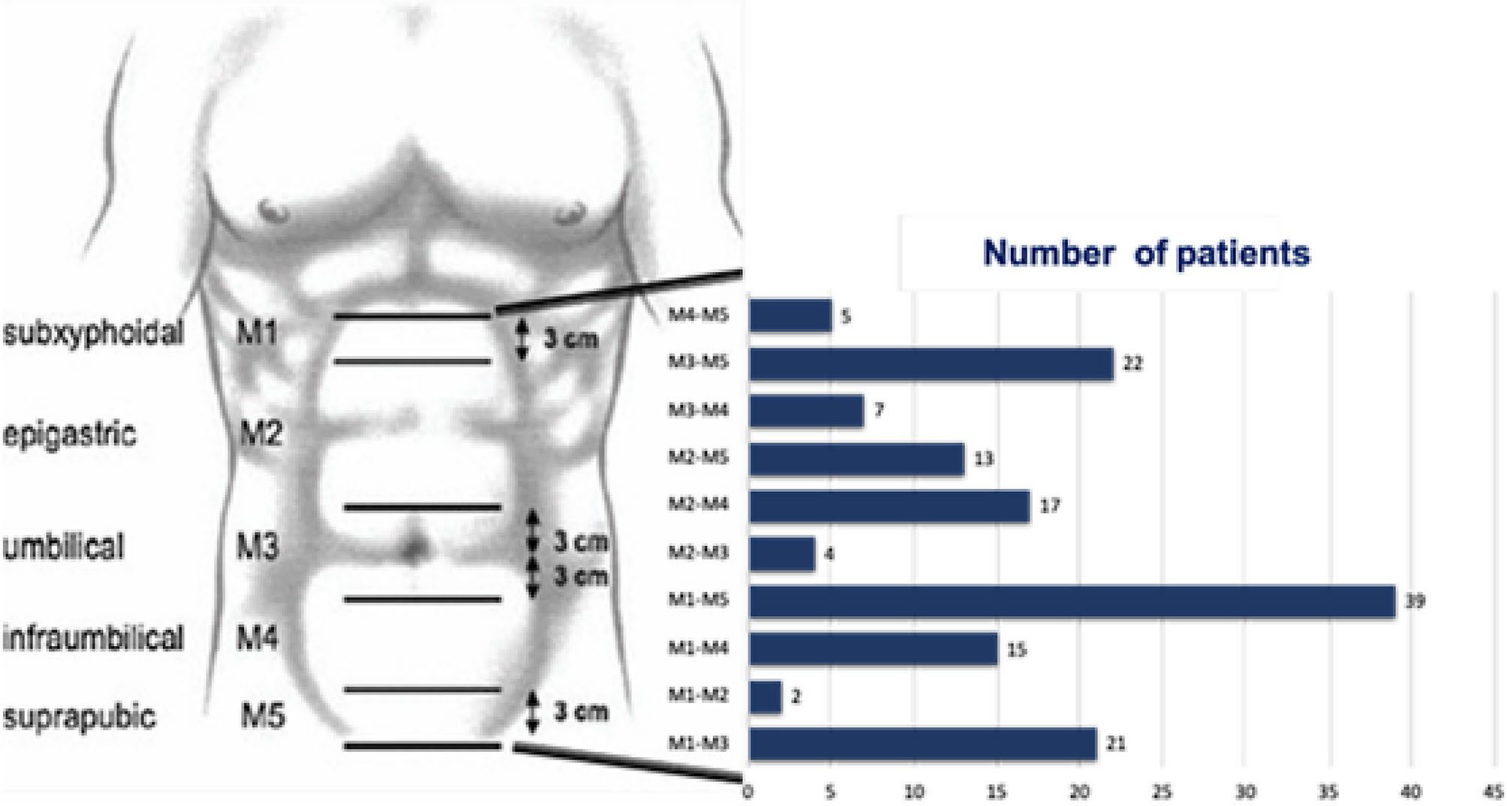
EHS Classification for Incisional Hernias

Fixation was performed using either traumatic methods—such as sutures, staples, and anchors—in 29.2% of cases, or atraumatic methods—using fibrin sealant and N-butyl-2-cyanoacrylate (NBCA)—in 72.5% of cases. A double-mesh technique (onlay-sublay) was required in 7.6% of the patients (Fig. 2).

**Table 2:** Technical and surgical characteristics of the patients

Variables	*N* = 171
Preoperative progressive pneumoperitoneum	6 (3.51%)
Use of botulinum toxin	50 (29.59%)
Preoperative antibiotic prophylaxis	169 (98.83%)
**Surgical approach**	
Laparoscopic	9 (5.29%)
Open	161 (94.71%)
**Type of clean surgery**	
Clean	167 (97.66%)
Clean-contaminated	4 (2.34%)
Defect width (cm) (mean ± SD)	10 (± 9.91)
Double mesh technique	13 (7.60%)
Anterior component separation	54 (31.58%)
Posterior component separation	117 (68.42%)
Rives technique	54 (31.58%)
Rives-Stoppa technique	40 (23.39%)
Transversus abdominis release	21 (13.60%)
Staged delayed closure	4 (2.34%)
Mesh use	165 (96.49%)
**Mesh position**	
Onlay	67 (40.00%)
Inlay	1 (0.60%)
Underlay	98 (59.30%)
**Mesh fixation**	
Traumatic fixation	50 (29.24%)
Atraumatic fixation	124 (72.51%)
**Panniculectomy type**	
Vertical panniculectomy	72 (42.11%)
Horizontal panniculectomy	63 (36.84%)

**SD**: Standard Deviation

To estimate the size of the adipose panniculus in relation to fixed anatomical structures, the Iglesias classification was used. In patients with morbid obesity—particularly those with a redundant panniculus (types II and III according to this classification)—significant surgical morbidity was observed, associated with pannus volume [10]. In 77.7% of cases, the panniculus extended beyond the inguinal ligament, and in 14.3%, it reached the mid-thigh.

**Fig. 3:**
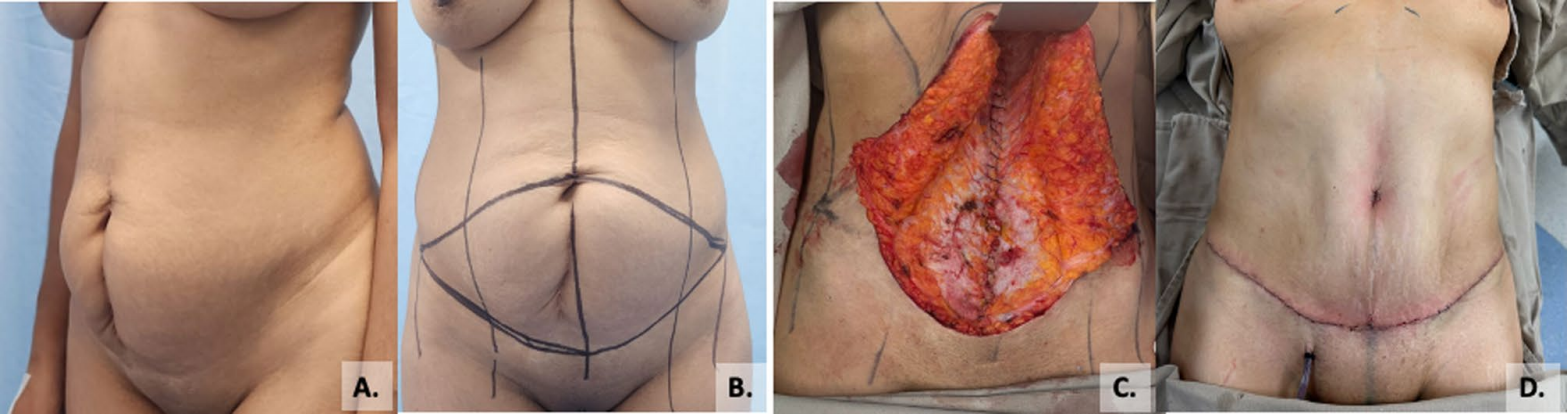
Patient with a ventral hernia classified as M2–M2W3–10 cm. Abdominal wall reconstruction was performed using the Rives-Stoppa technique combined with a horizontal panniculectomy to optimize both functional and aesthetic outcomes

**Fig. 4:**
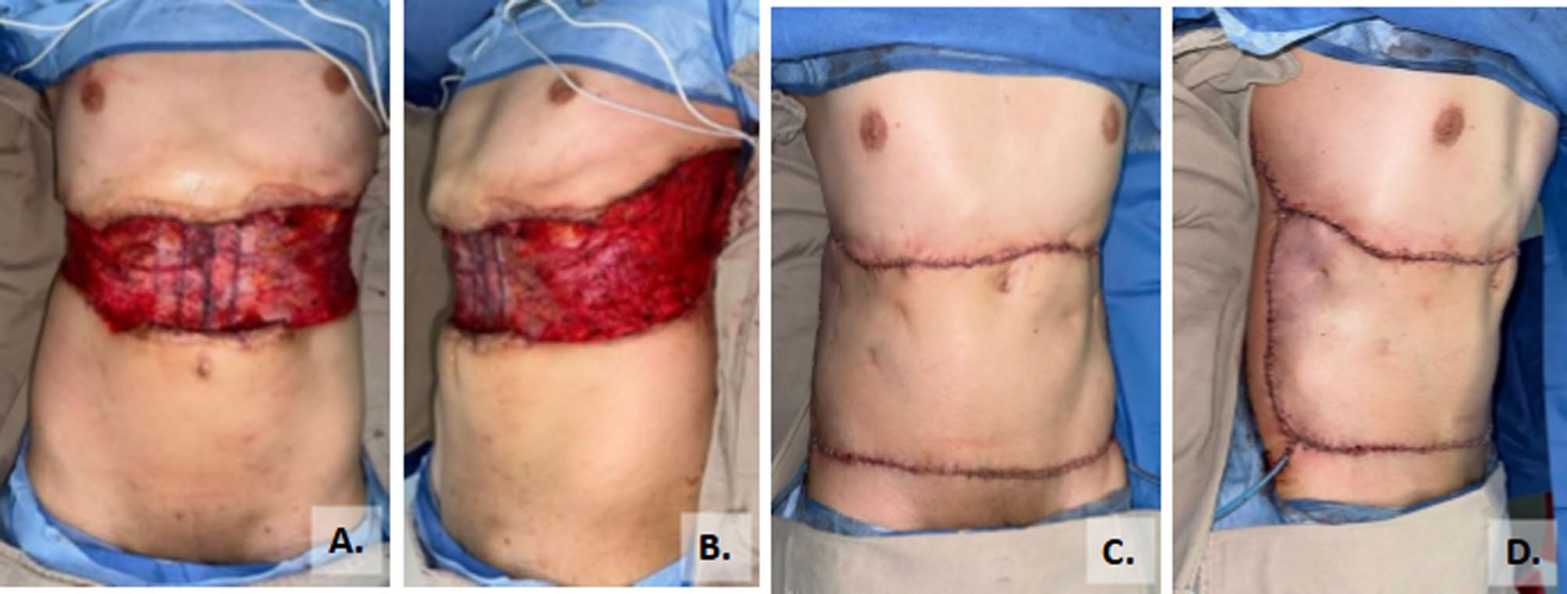
Abdominal wall reconstruction using anterior component separation and placement of a supra-aponeurotic mesh, combined with a Keystone flap

Vertical incision panniculectomy was performed in 42.1% of patients, while 18.1% underwent fleur-de-lis panniculectomy. Surgical drains were used in all cases, placed in the subcutaneous tissue (Fig. 3). In 2.2% of patients, management of the superficial layers of the abdominal wall required panniculectomy and reconstruction with Keystone flaps, as illustrated in the case shown in Fig. 4.

The average operative time was 155 min, with an average intraoperative blood loss of 100 ml. Following the procedure, 2.35% of patients required admission to the intensive care unit (ICU), and 5.26% underwent surgical reintervention. The median hospital stay was 2 days (RIC: 2.0–3.0), while 11.1% of patients were enrolled in an early discharge program, with follow-up through a home hospitalization program.

Regarding wound-related complications, seroma was observed in 4.68% of cases, hematoma in 2.9%, wound dehiscence in 12.87%, and enterocutaneous fistula in 1.75%. A total of 5.26% of patients required reintervention, and of these, 4% needed placement of a negative pressure wound therapy system for staged closure. No mortalities were reported Table 3.

The need for reintervention was statistically significantly associated with intraoperative bleeding (*p* = 0.014), skin dehiscence (*p* = 0.001), and a transverse defect diameter greater than 10 cm (*p* = 0.012).

In the analysis of factors associated with complications, no statistically significant differences were found regarding smoking, obesity, diabetes mellitus, body mass index, or defect size. The only variable significantly associated with the occurrence of complications was a longer hospital stay (*p* < 0.001). Furthermore, the analysis of mesh placement location (Onlay, Inlay, Underlay) showed no statistically significant association with the presence of surgical site infection (*p* > 0.05 for all comparisons).Table 4**and** Table 5.

**Table 3:** Intraoperative complications

Variables	*N* = 171
**Intraoperative Complications**	
Intestinal injury	5 (2.92%)
Intraoperative blood loss (cc) (mean ± SD)	100 (± 108.92)
Need for transfusion	1 (0.59%)
**Postoperative Complications**	
Need for ICU admission	4 (2.35%)
Use of drains in the postoperative period	160 (94.12%)
Length of hospital stay (days) (mean ± SD)	2 (± 3.34)
Readmission	13 (7.65%)
Surgical site infection (SSI)	13 (7.60%)
Hematoma	5 (2.92%)
Seroma	8 (4.68%)
Enterocutaneous fistula	3 (1.75%)
Skin dehiscence	22 (12.87%)
Use of negative pressure system	7 (4.09%)
Reintervention	9 (5.26%)
**Follow-up**	
Home Hospitalization Program (HHP)	19 (11.11%)
30-day complications	28 (16.37%)
***Clavien-Dindo Classification***	
*I*	*22 (57.89%)*
*II*	*7 (18.42%)*
*III*	*1 (2.63%)*
*IIIA*	*1 (2.63%)*
*IIIB*	*5 (13.16%)*

**SD**: Standard Deviation; **ICU**: Intensive Care Unit; **SSI**: Surgical Site Infection; **HHP**: Home Hospitalization Program

**Table 4:** Medical history and hospital stay according to complications

Variables	All	Yes	No	*p*-value
	*N* = 171	*N* = 34	*N* = 137	
Smoking	30 (18%)	6 (18%)	24 (18%)	> 0.9^a^
Diabetes mellitus	26 (15%)	5 (15%)	21 (15%)	> 0.9^a^
BMI (kg/m²), median (IQR)	27.8 (25.0, 30.5)	28.3 (25.3, 31.5)	27.7 (25.0, 30.4)	0.6^b^
Obesity	51 (30%)	12 (35%)	39 (28%)	0.4^a^
Defect size (cm), median (IQR)	10.0 (10.0, 14.0)	12.0 (10.0, 15.0)	10.0 (10.0, 14.0)	0.4^b^
Hospital stay (days), median (IQR)	2.0 (2.0, 3.0)	3.0 (2.0, 8.0)	2.0 (2.0, 3.0)	< 0.001^b^

^a^ Pearson’s chi-squared test; ^b^ Wilcoxon rank-sum test

**Table 5:** Variables and surgical site infection

Variables	All	Yes	No	*p*-value
	*N* = 171	*N* = 13	*N* = 158	
Onlay mesh placement	66 (39%)	6 (50%)	60 (38%)	0.5^a^
Inlay mesh placement	5 (2.9%)	0 (0%)	5 (3.2%)	> 0.9^a^
Underlay mesh placement	98 (58%)	8 (62%)	90 (57%)	0.8^a^

^a^ Pearson’s chi-squared test

## Discussion

Our study evaluated the outcomes of performing abdominal wall reconstruction and panniculectomy in a single surgical session, demonstrating that this strategy may be associated with limited morbidity. These findings support the safety and feasibility of a comprehensive approach in this complex patient population [13–16].

As the prevalence of obesity continues to rise, so too will the number of patients requiring abdominal wall reconstruction and panniculectomy. Performing panniculectomy during the same procedure aims to remove poorly vascularized tissue and resect previous scars, thereby reducing tension on the abdominal wall during hernia repair and minimizing the anesthetic risks associated with performing these procedures separately [14–18].

The findings of this study suggest that the concomitant performance of panniculectomy in patients undergoing abdominal wall reconstruction represents a valid therapeutic option. A significant association was found between defects larger than 10 cm, skin dehiscence, and intraoperative bleeding greater than 100 ml, and an increased risk of surgical reintervention. No statistically significant associations were found between mesh location, patient comorbidities, length of hospital stay, or the type of panniculectomy performed.

**Fig. 5:**
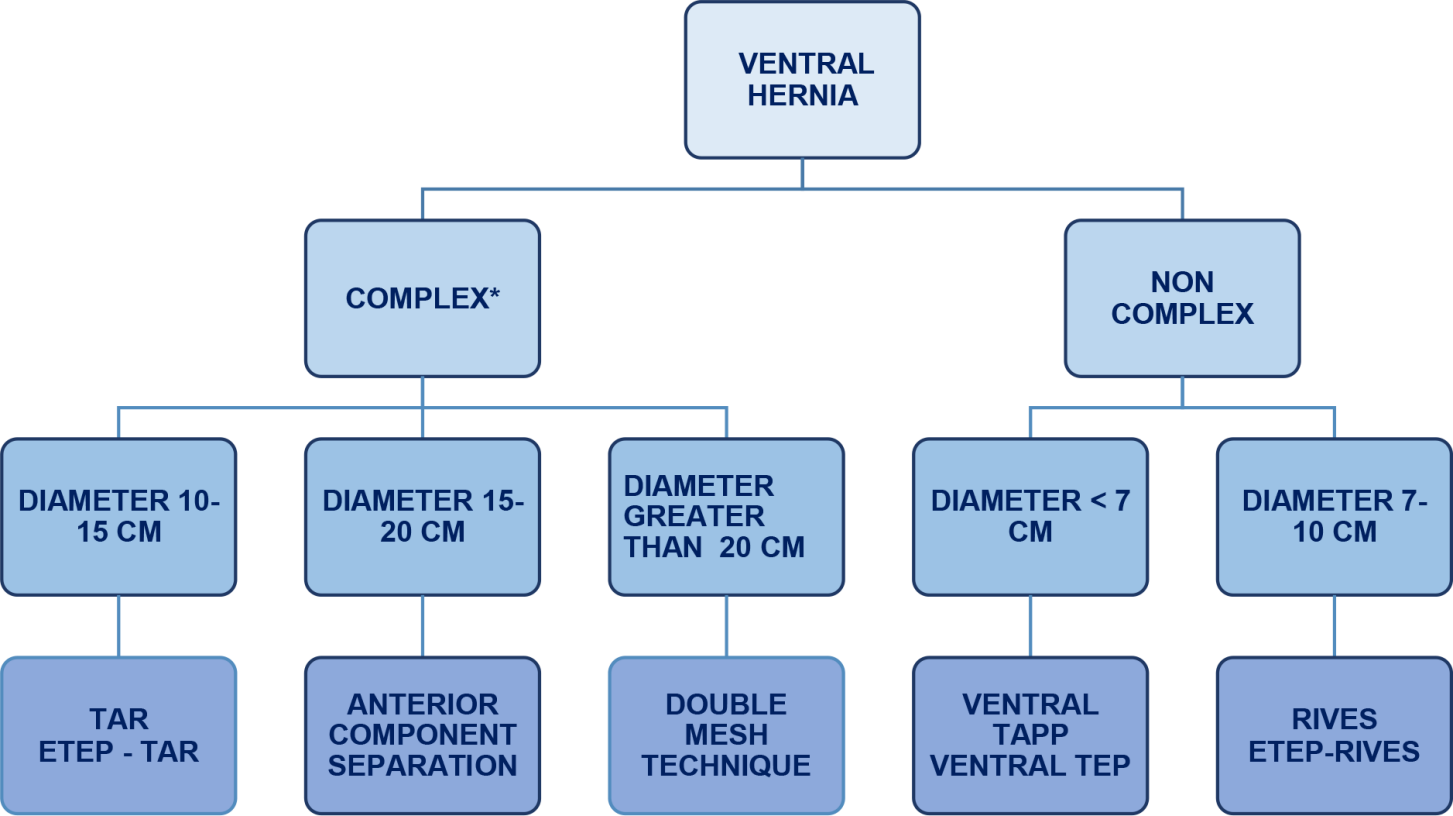
Surgical decision-making algorithm of our institution. ***TAPP*** transabdominal Preperitoneal. ***TAR*** Transversus abdominis muscle release. ***TEP*** Totally Extraperitoneal. ***ETEP*** Extended Totally Extraperitoneal” *A ventral hernia is considered complex when one or more of the following features are present: large defect size, generally greater than 10 cm in transverse diameter, loss of domain, multiple recurrences or prior repair failures, history of surgical site infection or presence of infected mesh, altered abdominal wall anatomy due to multiple surgeries, radiotherapy, or trauma, need for advanced reconstructive techniques

In our study, 96.5% of repairs involved the use of a medium-weight, large-pore synthetic polypropylene mesh. Regarding the techniques for midline restoration, 59.3% of cases used an underlay mesh position, followed by onlay in 40%, and inlay in 0.6%. A double-mesh technique was used in 7.6%, and no intraperitoneal meshes were implanted. Despite the inherent risks and potential complications associated with the surgical repair of complex ventral hernias, selecting the most effective approach to achieve complete closure of the anterior fascia is essential. To this end, we propose an algorithm that may facilitate the surgical strategy in such complex cases (Fig. 5). This classification is based on criteria adopted by international surgical societies such as the European Hernia Society (EHS) [19] and the Ventral Hernia Working Group (VHWG) [20], adapted to our institutional experience. Therefore, the term “complex ventral hernia” refers to those hernias that, due to their anatomical or clinical characteristics, involve greater technical difficulty for repair and require advanced reconstructive techniques, such as component separation, Transversus Abdominis Release (TAR), or the placement of double mesh.

In our group, most patients undergo prosthetic repair in the retromuscular (sublay) position, with a preference for posterior component separation techniques due to their demonstrated benefits: lower wound complication rates, reduced prosthetic exposure, and improved long- term functional integration. However, the magnitude and complexity of some hernia defects require an adapted approach. In cases where anatomical and functional restoration of the abdominal wall is not possible despite optimal preoperative optimization and posterior component separation, anterior component separation is employed. This allows repair of larger defects but is associated with a higher risk of surgical site complications (SSO). Moreover, in oncologic scenarios involving extensive abdominal wall resection and muscle loss, it may be unfeasible to conceal the prosthesis in the retromuscular space. In these contexts, advanced techniques such as dual mesh repair may be required, which sometimes necessitate placement of prosthetic material in the supra-aponeurotic (onlay) space, accepting a higher risk of wound complications.

Anterior component separation was performed in 31.5% of patients, while posterior component separation techniques were used in the remaining 68.4%. Retromuscular repair techniques were applied in 42.1% of cases; the most commonly used were the Rives technique (31.5%) and the Rives–Stoppa technique (23.3%). A delayed staged closure was required in 2.34% of cases. Follow-up was conducted at 60 days.

The majority of complications were mild (Grade I–II), not requiring invasive interventions. However, 15.8% of patients (Grades IIIA and IIIB) required additional procedures— a rate consistent with that reported in the literature for abdominal wall reconstruction procedures performed concomitantly with panniculectomy.The inherent complexity of these procedures, the use of prosthetic materials, and prolonged surgical times are all contributing factors, and globally they generate concern due to the associated high morbidity, increasing the difficulty of these surgeries. According to our findings, the inclusion of a concomitant panniculectomy did not represent an additional factor increasing the complication rate. It is worth noting that most of the recorded complications were of low severity (Clavien-Dindo < II), which supports this assertion. The documented outcomes may be attributed to the surgical team’s experience, collaborative work among involved professionals, appropriate case selection, and an institutional culture focused on patient safety.

These findings are consistent with reports in the literature. For example, Diaconu et al. [14] found a significantly higher prevalence of surgical site infection (SSI) when comparing simultaneous panniculectomy and abdominal wall reconstruction (35% vs. 21%; *p* = 0.018). Similarly, Fischer et al. [7], In the analysis of 55,537 patients from the ACS-NSQIP database, it was demonstrated that combining panniculectomy with open ventral hernia repair (VHR-PAN) is associated with a significantly increased risk of postoperative complications compared to hernia repair alone. Specifically, the combined procedure was linked to a 69% higher likelihood of wound complications, a 2.1-fold increased risk of surgical reintervention, a 2.5-fold increase in venous thromboembolism, and a twofold increase in overall medical morbidity (*p* < 0.001 in all cases, except VTE *p* = 0.044). These findings underscore the importance of careful patient selection and individualized risk-benefit assessment when considering combined surgical approaches. These findings are to be expected, considering that panniculectomy involves the creation of large flaps and extensive dissections, generating more dead space, which is associated with a high risk of SSI. However, these complications can often be resolved conservatively without increasing the reoperation rate.

Complex ventral hernias typically require an average hospital stay of 5.33 days [18]. In our study, the average hospital stay was 2.3 days. The implementation of a strict prehabilitation protocol, standardized surgical techniques, and intra- and post-operative multidisciplinary support (HPC) proved to be a key element in patient management, optimizing the use of resources. In this cohort, 11.1% of patients were eligible for outpatient follow-up, with no readmissions recorded within 30 days. No mortality was reported during the follow-up period. Preoperative optimization has emerged as a strategy to address modifiable patient risk factors in order to improve outcomes [19]. For this reason, all our patients undergo a multidisciplinary evaluation prior to surgery to enhance postoperative results.

These findings align with the literature on standardized care, the establishment of centers of excellence, and the centralization of complex surgeries — strategies that, in the field of abdominal wall surgery, are associated with better clinical outcomes, professional development of surgeons, and economic benefits for healthcare institutions.

The results demonstrate that the methodical and systematic implementation of a multidisciplinary collaborative work model — exemplified by the simultaneous performance of panniculectomy and abdominal wall reconstruction — is feasible in a specific healthcare setting, particularly when strategies inspired by Enhanced Recovery After Surgery (ERAS) protocols, already successfully applied in other surgical specialties, are adopted. To our knowledge, this study represents a pioneering contribution in the Latin American context, as no prior research on this combined approach has been published in the region.

The limitations of this study include its retrospective and observational nature, the absence of a control group, and the sample size. Regarding the 60-day follow-up period, it focused exclusively on the detection of early complications, such as surgical site occurrence (SSO), without including the assessment of long-term hernia recurrence. We acknowledge this limitation and emphasize that a comprehensive analysis of recurrences would require a study with extended clinical follow-up. Although the descriptive nature of the study implies limited generalizability, its strengths lie in the sample size, its innovative character within the Latin American context (with few existing studies on the subject), its reporting of complication rates similar to those previously described, and the implementation of a specialized and collaborative surgical team.

One of the main limitations of this study is the lack of validated tools to objectively assess quality of life, postoperative comfort, and patient satisfaction. Although relevant clinical benefits were identified, these patient-centered outcomes were not quantified using standardized instruments. Therefore, we recommend that future research, preferably with a prospective design, incorporate validated scales that allow for a more accurate measurement of these fundamental aspects, ensuring a comprehensive evaluation of surgical outcomes.

The clinical implications of this study suggest that a multidisciplinary management approach with favorable surgical outcomes for both patients and institutions is feasible within a specific context. Furthermore, the findings open the door for future research in an area of growing clinical interest. The simultaneous achievement of functional and aesthetic goals is attainable, but it must be part of a well-structured institutional strategy that considers the potential risk of higher-than-expected adverse outcomes.

## Conclusions

Our study suggests that combining abdominal wall reconstruction with panniculectomy is a feasible surgical strategy in selected patients, within the framework of a collaborative approach that systematically integrates preoperative, intraoperative, and postoperative interventions.

Factors such as a hernia defect with a transverse diameter greater than 10 cm, intraoperative bleeding exceeding 100 ml, and the occurrence of skin dehiscence were identified as variables associated with an increased risk of surgical reintervention.

## Electronic supplementary material

Below is the link to the electronic supplementary material.

Supplementary Material 1

Supplementary Material 2

## Declarations

### Use of artificial intelligence

The authors declare that no artificial intelligence-assisted technologies (such as large language models, chatbots, or image creators) were used in the production of this work.

## References

[1] Veilleux E, Lutfi R (2020) Obesity and ventral hernia repair: is there success in staging?? J Laparoendosc Adv Surg Tech Part A 30(8):896–899. 10.1089/lap.2020.0265 10.1089/lap.2020.0265 32453617

[2] Rahbari NN, Knebel P, Diener MK, Seidlmayer C, Ridwelski K, Stöltzing H, Seiler CM (2009) Current practice of abdominal wall closure in elective surgery - Is there any consensus? BMC Surg 9:8. 10.1186/1471-2482-9-8 19442311 10.1186/1471-2482-9-8 PMC2687428

[3] Dasari M, Wessel CB, Hamad GG (2016) Prophylactic mesh placement for prevention of incisional hernia after open bariatric surgery: a systematic review and meta-analysis. Am J Surg 212(4):615–622e1. 10.1016/j.amjsurg.2016.06.004 27659158 10.1016/j.amjsurg.2016.06.004

[4] den Hartog D, Dur AH, Tuinebreijer WE, Kreis RW (2008) Open surgical procedures for incisional hernias. Cochrane Database Syst Rev 2008(3):CD006438. 10.1002/14651858.CD006438.pub2 18646155 10.1002/14651858.CD006438.pub2 PMC8924951

[5] Dias Rasador AC, Marcolin P, da Silveira CAB, Kasakewitch JPG, Nogueira R, de Figueiredo SMP, Lima DL, Malcher F (2024) The impact of simultaneous panniculectomy in ventral hernia repair: a systematic review and meta-analysis. Hernia: J Hernias Abdom Wall Surg 28(6):2125–2136. 10.1007/s10029-024-03149-y 10.1007/s10029-024-03149-y 39240467

[6] Alli VV, Zhang J, Telem DA (2018) Impact of incisional hernia development following abdominal operations on total healthcare cost. *Surgical endoscopy*, *32*(5), 2381–2386. 10.1007/s00464-017-5936-8 Fischer, J. P., Tuggle, C. T., Wes, A. M., & Kovach, S. J. (2014). Concurrent panniculectomy with open ventral hernia repair has added risk versus ventral hernia repair: an analysis of the ACS-NSQIP database. *Journal of plastic, reconstructive & aesthetic surgery: JPRAS*, *67*(5), 693–701. https://doi.org/10.1016/j.bjps.2014.01.021 10.1016/j.bjps.2014.01.021 24525270

[7] Holland AM, Lorenz WR, Marturano MN, Hollingsworth RK, Scarola GT, Mead BS, Heniford BT, Augenstein VA (2024) Concurrent panniculectomy with abdominal wall reconstruction: A Propensity-scored matched study of quality improvement outcomes. Plast Reconstr Surg Glob Open 12(12):e6381. 10.1097/GOX.0000000000006381 39726817 10.1097/GOX.0000000000006381 PMC11671086

[8] Zemlyak AY, Colavita PD, Djouzi E, Walters S, Hammond AL, Hammond L, Tsirline B, Getz VB, S., Heniford BT (2012) Comparative study of wound complications: isolated panniculectomy versus panniculectomy combined with ventral hernia repair. J Surg Res 177(2):387–391. 10.1016/j.jss.2012.06.029 22795269 10.1016/j.jss.2012.06.029

[9] Iglesias M, Butron P, Abarca L, Perez-Monzo MF, de Rienzo-Madero B (2010) An anthropometric classification of body contour deformities after massive weight loss. Ann Plast Surg 65(2):129–134. 10.1097/SAP.0b013e3181c9c336 20606587 10.1097/SAP.0b013e3181c9c336

[10] McNichols CHL, Diaconu S, Liang Y, Ikheloa E, Kumar S, Kumar S, Nam A, Rasko Y (2018) Outcomes of ventral hernia repair with concomitant panniculectomy. Ann Plast Surg 80(4):391–394. 10.1097/SAP.0000000000001277 29309330 10.1097/SAP.0000000000001277

[11] Khansa I, Janis JE (2018) Management of skin and subcutaneous tissue in complex open abdominal wall reconstruction. Hernia: J Hernias Abdom Wall Surg 22(2):293–301. 10.1007/s10029-017-1662-3 10.1007/s10029-017-1662-3 28871371

[12] Gossett AG, Leavitt JD, Hooks WB 3rd, Hope WW (2025) Outcomes after ventral hernia repair with concurrent panniculectomy: A large database review. J Am Coll Surg 240(4):530–535. 10.1097/XCS.0000000000001287 10.1097/XCS.0000000000001287 39817658

[13] Diaconu SC, McNichols CHL, AlFadil S, Liang Y, Bai J, Silverman RP, Grant MP, Nam AJ, Rasko YM (2019) Postoperative outcomes in obese patients that undergo ventral hernia repair versus ventral hernia repair with concurrent panniculectomy. Plast Reconstr Surg 143(4):1211–1219. 10.1097/PRS.0000000000005471 30676508 10.1097/PRS.0000000000005471

[14] Shubinets V, Fox JP, Tecce MG, Mirzabeigi MN, Lanni MA, Kelz RR, Dumon KR, Kovach SJ, Fischer JP (2017) Concurrent panniculectomy in the obese ventral hernia patient: assessment of short-term complications, hernia recurrence, and healthcare utilization. J Plast Reconstr Aesthetic Surgery: JPRAS 70(6):759–767. 10.1016/j.bjps.2017.01.001 28286040 10.1016/j.bjps.2017.01.001

[15] Senchenkov A, Moran SL, Petty PM, Knoetgen J 3rd, Tran NV, Clay RP, Bite U, Johnson CH, Barnes SA, Sim FH (2009) Soft-tissue reconstruction of external hemipelvectomy defects. Plast Reconstr Surg 124(1):144–155. 10.1097/PRS.0b013e3181a80557 10.1097/PRS.0b013e3181a80557 19568053

[16] Hutchison CE, Rhemtulla IA, Mauch JT, Broach RB, Enriquez FA, Hernandez JA, Messa CA, Williams NN, Harbison SP, Fischer JP (2019) Cutting through the fat: a retrospective analysis of clinical outcomes, cost, and quality of life with the addition of panniculectomy to ventral hernia repair in overweight patients. Hernia: J Hernias Abdom Wall Surg 23(5):969–977. 10.1007/s10029-019-02024-5 10.1007/s10029-019-02024-5 31420773

[17] Ayyala HS, Weisberger J, Le TM, Chow A, Lee ES (2020) Predictors of discharge destination after complex abdominal wall reconstruction. Hernia: J Hernias Abdom Wall Surg 24(2):251–256. 10.1007/s10029-019-02054-z 10.1007/s10029-019-02054-z 31624964

[18] Fafaj A, de Figueiredo SMP, Rosen MJ, Petro CC (2024) Preoperative optimization in hernia surgery: are we really helping or are we just stalling? Hernia: J Hernias Abdom Wall Surg 28(3):925–930. 10.1007/s10029-024-02962-9 10.1007/s10029-024-02962-9 PMC11249412 38578363

[19] Capoccia Giovannini S, Podda M, Ribas S, Montori G, Botteri E et al (2024) What defines an incisional hernia as ‘complex’: results from a Delphi consensus endorsed by the European hernia society (EHS). Br J Surg 111(1):znad346. 10.1093/bjs/znad346 37897716 10.1093/bjs/znad346

[20] HerniaSurge Group (2018) International guidelines for groin hernia management. Hernia: J Hernias Abdom Wall Surg 22(1):1–165. 10.1007/s10029-017-1668-x 10.1007/s10029-017-1668-x PMC5809582 29330835

