# Supplementary material for: Clinical and perioperative outcomes of abdominal wall reconstruction and panniculectomy in a single surgical procedure: experience from a high-complexity center in bogotá, Colombia
Source: Hernia. 2025 Jul 21;29(1):236. doi: 10.1007/s10029-025-03415-7 (PMC12279593; doi:10.1007/s10029-025-03415-7)
Supplement: Supplementary file 2 — Supplementary Material 2 [file 10029_2025_3415_MOESM2_ESM.pdf]

SPRINGER NATURE | Springer

Consent form for Publication

I Carolina Riscanevo Bobadilla give my consent for information about myself/my child or ward/my relative (circle as appropriate) to be published in Hernia -The World Journal of Hernia and Abdominal Wall Surgery

Manuscript : Challenges and Advances in Abdominal Wall Hernia Repair in Obese Patients at a High-Complexity Center in Bogotá, Colombia: Towards a Safer and More Effective Surgery

Corresponding author: Carolina Riscanevo Bobadilla

NB: In cases where the patient has died or is incapable of giving consent, consent may be given by the next of kin. If the patient is under the age of 16, consent should be given by a parent or guardian.

I understand that the text and any pictures or videos published in the article

1. will be used only in educational publications intended for professionals
   Or
2. if the publication or product is published on an open access basis, I understand that it will be freely available on the internet and may be seen by the general public.

The pictures, videos and text may also appear on other websites or in print, may be translated into other languages or used for commercial purposes.

I understand that the information will be published without my/my child or ward's/my relative's (circle as appropriate) name attached, but that full anonymity cannot be guaranteed.

I have been offered the opportunity to read the manuscript.

I acknowledge that it is not possible to ensure complete anonymity, and someone may be able to recognize me. However, by signing this consent form I do not in any way give up, waive or remove my rights to privacy. I may revoke my consent at any time before publication, but once the information has been committed to publication ("gone to press"), revocation of the consent is no longer possible.

Name Carolina Riscanevo Bobadilla Date February 19, 2025

Signed CarolinaRiscanevo.

Author name Carolina Riscanevo Bobadilla Date February 19, 2025

Signed CarolinaRiscanevo.

Please keep this consent form in the patient's case files. The manuscript reporting this patient's details should state that 'Written informed consent for publication of their clinical details and/or clinical images

was obtained from the patient/parent/guardian/ relative of the patient. A copy of the consent form is available for review by the Editor of this journal.
